# Circulating adipokines are associated with Kawasaki disease

**DOI:** 10.1186/s12969-018-0243-z

**Published:** 2018-05-08

**Authors:** Xin-yan Zhang, Ting-ting Yang, Xiu-fen Hu, Yu Wen, Feng Fang, Hui-ling Lu

**Affiliations:** 1 Tongji Hospital, Tongji Medical College, Huazhong University of Science and Technology, Wuhan, 430000 Hubei China; 2 Department of Pediatrics, Tongji Hospital, No.1095. Jiefang Road, Qiaokou District, Wuhan City, 430000 Hubei Province China

**Keywords:** Adiponectin, Chemerin, Kawasaki disease, Omentin-1

## Abstract

**Background:**

The pathogenesis of Kawasaki disease are still not well understood. It was designed to investigate the relationship between adipokines including chemerin, omentin-1, adiponectin and acute Kawasaki disease.

**Methods:**

Enzyme-linked immunosorbent (ELISA) was used to detect serum levels of chemerin, omentin-1, adiponectin, and inflammatory cytokines IL-1β and TNF-α in 80 cases of patients diagnosed with Kawasaki disease (KD). In addition, 20 cases of children with fever and 20 cases of healthy children were selected as febrile and normal controls.

**Results:**

(1) Serum levels of chemerin in KD group (87.736 ± 56.310) are higher than that of both the healthy (41.746 ± 10.824) and the febrile controls (59.683 ± 18.282) (*P* < 0.01). (2) Circulating omentin-1 levels in Kawasaki disease group (389.773 ± 238.611) are significantly lower than that of febrile control (542.075 ± 177.995) (*P* < 0.01), also serum adiponectin levels in Kawasaki disease group (16.400 ± 12.243) reduced obviously compared with the febrile control group (35.074 ± 12.486). (3)Serum cytokine levels of IL-1β in Kawasaki disease group (13.656 ± 31.151) are higher than those of normal controls (2.415 ± 6.313) (*P* < 0.05). (4) Correlation analysis indicates that serum levels of chemerin are positively correlated with omentin-1 (*r* = 0.224, 95% CI 0.06–0.529, *P* < 0.05). Further, serum omentin-1 levels and total cholesterol (TC) are positively correlated (*r* = 0.358, 95% CI 0.169–0.518, *P* < 0.01).

**Conclusions:**

Circulating chemerin increased significantly in the acute stage of Kawasaki disease, while omentin-1 and adiponectin levels are decreased. These adipokines are closely associated with the early inflammation and lipid metabolism disorders of acute Kawasaki disease.

## Background

Kawasaki disease(KD) is an acute febrile illness, known as mucocutaneous lymph node syndrome, which mainly occurs in boys under 5 years old [1]. Cardiovascular manifestations are the main complications of KD such as coronary artery abnormalities, myocarditis, pericarditis, pericardial effusion, valvular dysfunction, left ventricular dysfunction, and arrhythmias [2]. And KD is the most common cause of coronary artery aneurysms (CAA) in children or young adults and the leading cause of acquired heart disease [3]. However, treatment of intravenous immunoglobulin (IVIG) plus acetylsalicylic acid (ASA) reduces the prevalence of coronary artery abnormalities from 32 to 50% to approximately 4% [4]. Although the pathogenesis of KD is still not fully understood, innate and specific immunity are always fully activated during the acute stage of KD, in which neutrophils, CD8 + T cells, dendritic cells, and macrophages are also activated successively in infiltration of artery walls [5, 6], then leading to the activation of the nuclear transcription factor NF-κB in monocytes macrophages [7]. This promotes the production of inflammatory cytokines such as IL-6 and TNF-α [8], further infiltrating vascular endothelium and causing immune activation.

Adipose tissue is not only a simple energy metabolism organ but also an important endocrine organ that could secrete numerous of proinflammatory cytokines such as TNF, IL-6, MCP1, leptin, and others [9, 10]. Also, it can secrete a series of anti-inflammatory adipokines including the CTRP family and Sfrp5, which play crucial protective roles in the inflammation and atherosclerosis [11, 12]. Adipokines are these pleiotropic molecules mainly secreted by adipocytes. It demonstrated that classical adipokines leptin, adiponectin and resistin play a major role in energy metabolism, inflammation, obesity, diabetes, cardiovascular disease and autoimmune diseases [13–16]. Recently, it has been conducted that adipokines including adiponectin, leptin, resistin, and visfatin were involved in the acute stage of KD and may participate in its progress of coronary artery lesions [17–19]. Therefore, it interests us that whether the more recently identified chemerin, and omentin, play roles in Kawasaki disease and whether they are associated with lipid metabolism disorders and coronary artery abnormalities of KD. In this study, we focus on the anti-inflammatory and pro-inflammatory effects of chemerin,omentin-1, and adiponectin on the acute phase of KD. Fortunately, it showed that adipokines including chemerin, omentin-1 and adiponectin involved in inflammation of acute KD and might be associated with its lipid metabolism disorders in this study.

## Methods

### Patients and groups

Eighty children diagnosed with Kawasaki disease (49 boys and 31 girls) in hospital from August 2015 to March 2017 and exclude other severe diseases were selected as KD group. This study was completed in April 2017. Inclusion criteria: (1) KD was accurately diagnosed according to the diagnose criteria of KD; (2) KD with coronary artery abnormalities and without coronary artery abnormalities of untreated KD cases were confirmed by heart echocardiography; (3) Clinical and laboratory examination data are completed. Exclusion criteria: (1) Sepsis cases with positive blood culture; (2) Accompanied by other cardiovascular or hypertension diseases, and primary disease associated with tumors, hematological diseases, congenital malformations, genetic metabolic diseases, primary myocarditis, primary diseases of major organs; (3) Relapsed patients have been treated; (4) Clinical and laboratory examination data are incomplete. A classification of coronary artery abnormalities based solely on Z score has been proposed in the new guidelines of 2017 [4]. Z-score classification has been adapted and recommended as follows: (1) No involvement: Always < 2; (2) Dilation only: 2 to < 2.5; or if initially< 2, a decrease in Z score during follow-up ≥1; (3) Small aneurysm: ≥2.5 to 5; (4) Medium aneurysm: ≥5 to ≤10, and absolute dimension < 8 mm; (5) Large or giant aneurysm: ≥10, or absolute dimension ≥8 mm. In this study, there were 24 cases of KD patients with coronary artery abnormalities who were enrolled in the CAL group and other fifty-six children were NCAL group. It must be emphasized that all children with coronary artery abnormalities in this study showed coronary dilatation. In addition, twenty children with general fever and respiratory infections children who were admitted to our hospital at the same time (11cases of pneumonia and 9 cases of bronchitis) were selected as the febrile control group. In addition, 20 children in the healthy group who had a physical examination in our hospital were selected as normal control. It should be noted that these children are all about to enter kindergarten before which they have to conduct a physical examination. And in China, only 3 years old can attend kindergarten. This is the reason why the average age of these healthy children is 3 years old. All patients diagnosed with KD treated with intravenous immunoglobulin (IVIG) (2 g/kg given as a single intravenous infusion) and acetylsalicylic acid (ASA, 30 mg·kg^− 1^·d^− 1^) within the first 10 days of illness. IVIG resistance means that patients with KD have the persistent or recurrent fever after primary therapy with IVIG plus ASA [4]. The diagnosis of typical Kawasaki disease met the diagnostic criteria proposed in the 2017 AHA guidelines [4], in which children with a history of more than 4 days of fever have at least the following four major clinical manifestations, including skin rash, lymphadenectasis, bilateral conjunctival congestion, oral changes, hard swollen and molting of fingertips. Incomplete Kawasaki disease means fever for 5 days or more than 5 days plus only two or three major clinical manifestations, and excludes a diagnosis of scarlet fever, drug hypersensitive syndrome, Stevens-Jonson syndrome, toxic shock syndrome, adenovirus infections, Epstein-Barr febrile illness (BB) and virus infection febrile illness. According to this, there were fourteen incomplete KD and sixty-six complete KD patients in this study.

### Methods

#### Serum adipocytokines chemerin, omentin-1, adiponectin, and cytokines TNF-α and IL-1β detection

Peripheral blood of 2 mL in heparin anticoagulation tube was collected in the acute stage of KD (usually 1-11 days during the course of the disease) prior to receiving IVIG treatment. Blood was also obtained from children in the febrile group prior to receiving treatment and in the healthy group. Blood was centrifuged and serum immediately saved in − 80 °C for later analysis. Enzyme-linked immunosorbent (ELISA) was used to detect serum levels of chemerin, omentin, adiponectin and the inflammatory cytokines IL-1β and TNF-α. Chemerin ELISA kit was supplied by RND operation (DCMHOO), omentin-1 ELISA kit was supplied by Biovender operation (RD191100200R), TNF-α (557966) and IL-1β (550610) ELISA kit were supplied by BD operation and adiponectin ELISA kit was supplied by Ebioscience operation (BMs2032/2). A Siemens automatic enzyme table analyzer(9659) was used for analysis. Serum levels of adipokines including chemerin, omentin, and adiponectin were detected at the same time, as well as cytokines TNF-α and IL-1β. All assays were performed in accordance with the kit instructions, with detection at 450 nm wavelength using the standard curve to calculate the concentrations.

#### Biochemical indicators, echocardiography, and electrocardiogram

Levels of serum IL-6, high sensitivity c-reactive protein (CRP), erythrocyte sedimentation rate (ESR), Procalcitonin (PCT), NT-proBNP, blood lipid and other parameters in KD patients prior to receiving treatment were performed using an automated biochemical analyzer in the hospital Clinical Biochemistry Laboratory. Heart echocardiography and electrocardiogram were performed by the cardiac function department and the pediatric cardiovascular laboratory respectively.

### Statistics analysis

SPASS 20.0 software was used for to statistical processing. Normally distributed continuous data were expressed as the mean ± standard deviation. Comparisons of frequencies between groups were analyzed by t-tests or one-way ANOVA analysis and differences among groups were assessed using chi-square tests. Correlation between different adipokines and other biochemical indicators were analyzed by Pearson or Spearman analysis. The significance of difference was calculated by Scheffe’s test and a *P* value less than 0.05 was considered statistically significant.

## Results

### Clinical characteristic of patients with KD

Among eighty patients with KD, there are forty-nine boys and thirty-one girls, whose average age is (2.994 ± 2.267) years. There are no significant differences between KD and the febrile and healthy controls (*P > 0.05*). All children in KD group have treated with IVIG within 10 days of hospital admittance. The CAL group, composed of KD patients with coronary artery dilatation, including 24 cases (30%) and accordingly the NCAL group consists of 56 cases (70%). Of eighty cases of KD, a total of fourteen children (17.5%) are diagnosed with incomplete KD and sixty-six patients (82.5%) for typical KD. It’s noteworthy that this percentage doesn’t represent the prevalence of coronary artery abnormalities and incomplete KD in KD. It just reflects the ratio of CAL or incomplete and KD. The inflammatory markers in the acute stage of KD including CRP, ESR, and PCT are higher than the febrile group (*P < 0.05*). Also, it shows that blood cholesterol and high-density lipoprotein (HDL) is lower than febrile controls (*P < 0.05*) in the acute phase of KD. Notably, NT-proBNP clearly increased (1326.462 ± 2185.425) and then reduced after treating with IVIG 175.156 ± 115.665) *(P < 0.05)*. KD patients with CAL (1831.676 ± 2048.909) has higher levels of NT-proBNP compared with NCAl group (1083.960 ± 2247.476), though there are no significant differences *(P > 0.05)*, which might due to limited cases of KD in this study. Similarly, patients with typical KD have relative higher NT-proBNP levels compared with patients with incomplete KD. But there’s no significant statistical difference *(P > 0.05),* which remains to be further confirmed by larger samples (Tables 1, 2, and 3).

**Table 1:** General characteristics of patients with Kawasaki disease

	Gender (M/F)	Age	BMI	Fever time (day)
KD (*n* = 80)	49/31	2.994 ± 2.267	15.0923 ± 1.358	6.788 ± 3.393
Febrile controls (*n* = 20)	12/8	3.278 ± 3.656	16.303 ± 2.413	2.650 ± 2.277
Healthy controls (*n* = 20)	9/11	3.00 ± 0.0	15.153 ± 2.993	0.000
F	–	0. 118	1.837	5.161
P	–	0.889	0.168	< 0.001^*^

*represents a significant difference between groups, which is *P* < 0.05

**Table 2:** General characteristics and biochemical indicators of patients with Kawasaki disease

	KD group (*n* = 80)	Febrile controls (*n* = 20)	t	*P*
CRP mg/L	76.465 ± 52.761	17.100 ± 27.852	−6.601	< 0.001^*^
ESR mm/H	53.877 ± 26.773	11.583 ± 7.914	−10.493	< 0.001^*^
IL-6 pg/mL	153.578 ± 331.333	5.900 ± 6.494	−3.803	< 0.001^*^
PCT ng/L	1.359 ± 3.364	0.135 ± 0.165	−3.153	0.002^*^
HB g/L	106.526 ± 10.517	117.588 ± 12.430	3.790	< 0.001^*^
PLT *10^3/L	350.434 ± 125.649	327.588 ± 128.848	−0.675	0.502
TG mmol/L	1.208 ± 0.641	1.287 ± 0.601	0.400	0.690
TC mmol/L	3.129 ± 0.619	3.562 ± 0.591	2.639	0.010^*^
HDL mmol/L	0.613 ± 0.240	1.146 ± 0.309	−6.269	< 0.000^*^
LDL mmol/L	1.974 ± 0.562	1.994 ± 0.565	−0.103	0.918

*represents a significant difference between groups, which is *P* < 0.05

**Table 3:** Biochemical indicators of patients with Kawasaki disease prior treatment and posttreatment of IVIG

	Prior treatment of IVIG	Posttreatment of IVIG	t	*P*
CRPmg/L	81.726 ± 56.671	4.014 ± 4.678	8.424	< 0.001^*^
ESR mm/H	54.290 ± 26.752	41.207 ± 29.089	1.815	0.075
IL-6 pg/mL	157.145 ± 298.991	3.876 ± 3.032	2.989	0.005^*^
PCT ng/L	1.982 ± 4.540	0.121 ± 0.131	2.493	0.017^*^
HB g/L	105.486 ± 8.688	108.841 ± 10.696	−1.473	0.145
PLT *10^3/L	330.432 ± 128.981	497.694 ± 141.631	−5.278	< 0.001^*^
NF-proBNP pg/mL	1326.462 ± 2185.425	175.156 ± 115.665	3.199	0.003^*^
TG mmol/L	1.296 ± 0.844	1.565 ± 0.606	−1.239	0.220
TC mmol/L	3.057 ± 0.623	3.571 ± 1.109	−2.526	0.014^*^
HDL mmol/L	0.613 ± 0.240	0.740 ± 0.215	−1.841	0.071
LDL mmol/L	1.973 ± 0.562	2.343 ± 0.480	−2.177	0.035^*^
Apo-A1 g/L	0.655 ± 0.249	0.943 ± 0.253	−3.311	0.002^*^
Apo-B g/L	0.697 ± 0.202	0.681 ± 0.094	0.341	0.753

*represents a significant difference between groups, which is *P* < 0.05

### The serum levels of adipokines including chemerin, omentin-1 and adiponectin in the acute stage of KD, general fever and healthy controls

Circulating chemerin in KD group (87.736 ± 56.310) is higher than that of febrile (59.683 ± 18.282, *P* < 0.01) and normal controls (41.75 ± 10.833, *P* < 0.01). It doesn’t show any significant differences between KD with CAL (82.584 ± 48.745) and NCAL (89.944 ± 59.534) in the serum levels of chemerin (*P > 0.05*). By contrast, the levels of omentin-1 in acute KD (389.773 ± 238.611) is lower than general febrile control (542.08 ± 177.995, *P < 0.01*). There are no significant differences between KD and healthy control (385.65 ± 105.535, *P > 0.05*), which might because of limited samples and individual variation. In the acute stage of KD, adiponectin levels (16.400 ± 12.243) decreass compared with the general febrile patients (35.074 ± 12.486, *P < 0.05*). There are no significant differences compared with the healthy children (16.959 ± 7.576) (*P > 0.05*). In addition, we didn’t find serum levels of both omentin-1 and adiponectin show any significant differences between KD with CAL and NCAL (*P > 0.05*) (Tables 4 and 5).

**Table 4:** Serum levels of adipokines in the acute phase of KD and control subjects

Groups	Chemerin (ng/mL)	Omentin-1 (ng/mL)	Adiponectin (ug/mL)
Healthy controls(*n* = 20)	41.746 ± 10.824	385.662 ± 105.547	16.959 ± 7.576
Febrile controls(*n* = 20)	59.683 ± 18.282	542.075 ± 177.995	35.074 ± 12.486
KD group(*n* = 80)	87.736 ± 56.310	389.773 ± 238.611	16.400 ± 12.243
*P*	< 0.001^*^	0.016^*^	< 0.001^*^

*represents a significant difference between groups, which is *P* < 0.05

**Table 5:** Comparison of serum adipokines levels between CAL and NCAL group in KD patients

Groups	CAL (*n* = 24)	NCAL (*n* = 56)	t	*P*
Chemerin (ng/mL)	82.584 ± 48.745	89.944 ± 59.534	−0.533	0.595
Omentin-1(ng/mL)	410.312 ± 258.103	407.651 ± 267.921	0.041	0.967
Adiponectin(ug/mL)	20.483 ± 17.349	16.891 ± 14.820	0.943	0.348
TNF-α (pg/ml)	14.678 ± 12.402	9.445 ± 6.024	1.393	0.187
IL-1β (pg/ml)	9.299 ± 20.718	15.523 ± 34.677	−0.817	0.416

*represents a significant difference between groups, which is *P* < 0.05

### Levels of cytokines IL-1β and TNF-α in the acute stage of KD, general fever and healthy controls

**Fig. 1:**
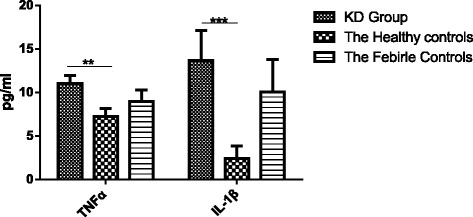
Serum levels of cytokines in the acute phase of KD and control subjects. This figure shows that the levels of TNF-α in KD group were obviously higher than that of the healthy group *(P < 0.05).* Also, IL-1β levels in KD group were significantly increased compared with healthy group *(P < 0.05).* **Represents a significant difference between groups, which is *P* < 0.05. ***Represents a significant difference between groups, which is *P* < 0.01.

Circulating TNF-α in acute KD (11.015 ± 8.626) is higher than that of healthy control (7.261 ± 4.056, *P < 0.05).* Similarly, IL-1β levels in patients with KD (13.656 ± 31.151) are significantly increased compared with healthy children (2.415 ± 6.313, *P < 0.05),* but there are no significant differences between KD group and febrile control (*P > 0.05*, Fig. 1).

### Correlation analysis of adipokines and other parameters

**Fig. 2:**
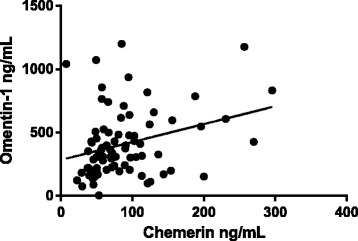
Chemerin is positively correlated with omentin (*r* = 0.224, *P* < 0.05)

**Fig. 3:**
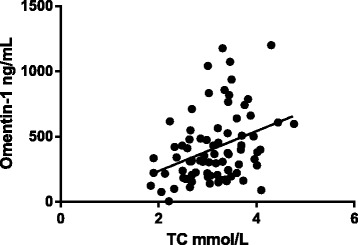
Omentin-1 levels and TC are positively correlated (*r* = 0.358, *P* < 0.05)

Through correlation analysis, it shows chemerin is positively correlated with omentin-1 (*r* = 0.224, 95% CI 0.06–0.529, *P* < 0.05). Further, total cholesterol (TC) is positively correlated with omentin-1 (*r* = 0.358, 95% CI 0.169–0.518, *P* < 0.05), which implied that adipokines especially omentin-1 are associated with disorders of lipid metabolism (Figs. 2 and 3).

## Discussion

As recently discovered adipokines, chemerin, omentin-1, and adiponectin play crucial roles in inflammatory response and are closely involved in cardiovascular diseases. Takeshida et al. [20] reported that adiponectin levels were significantly higher in KD patients; Nozue et al. [21] also found resistin levels elevated in KD but its concentrations were unlikely to predict the prognosis of the disease in the acute stage; Liu et al. [22] published data suggesting that leptin participates in the systemic inflammatory response but with controversial results; Kim et al. [18] found that resistin is significantly higher in KD patients, although it has no prognostic value to predict coronary anomalies in the acute stage.

This study aimed to evaluate the levels of these three adipokines in the acute phase of KD and to investigate the associations between adipokines and coronary artery abnormalities or lipid metabolism disorders of KD. Our research demonstrated that (1) circulating chemerin levels are increased in the acute stage of KD. (2)By contrast, omentin-1 and adiponectin levels are decreased compared with general fever patients in the acute stage of KD. (3)Furthermore, cytokine IL-1β is elevated in patients with acute KD, which is similar to inflammatory cytokines such as TNF-α. On the other hand, IL-1β has a decreasing trend in CAL compared with the NCAL group, which might imply that IL-1β involved in the process of inflammatory infiltration and immune vasculitis in the acute phase of KD. Moreover, (4) we find that circulating omentin-1 is positively correlated with both chemerin and total cholesterol, which suggests that chemerin and omentin-1 are involved in the lipid metabolism disorders in the acute stage of KD.

Chemerin was identified as a cDNA sequence called TIG2 (Tazarotene-induced gene 2) in 1997 firstly [23], whose expression was up-regulated during the treatment of psoriatic lesion by tazarotene, which is a kind of retinoic acid receptors (RARs). It has been demonstrated that the chemerin as a type of adipokine, whose gene expression and its receptor, chemerin-like receptor1 (CMKLR1), was significantly higher in adipose tissue of obese models through a signal sequence trap in 2007 in the first place [24].Our study firstly indicated that circulating chemerin levels increased significantly in the acute stage of KD, which were associated with its effects on the early phase of immune response and inflammatory reaction. Chemerin could modulate immune responses through its chemotactic effects and accumulation of antigen-presenting cells including macrophages and dendritic cells at the sites of damage areas [25, 26]. Several studies have reported that chemerin was closely associated with the inflammatory response related to obesity, metabolic syndrome, rheumatoid arthritis and cancer [27–30]. Chemerin receptor CMKRL1 expressed in human vascular endothelial cells could bind to chemerin, which induces inflammation and angiogenesis processes [31, 32]. In addition, chemerin was involved in the development of inflammation in cardiovascular disease and atherosclerosis [33–35], and circulating chemerin were associated with soluble ICAM-1 and E-selectin [36], which provide the greatest evidence regarding endothelial-cell activation that could trigger vascular inflammation. We didn’t find a direct relationship between circulating chemerin and KD with coronary artery abnormalities, or incomplete KD. This may be explained that chemerin was mainly involved in the process of immune regulation and inflammatory activation in acute KD, also might be associated with the inflammatory infiltration of vascular endothelial cells. However, the pathogenesis of chemerin in coronary artery lesions of patients with KD still need to be confirmed further.

Omentin-1 or interlectin-1, a new cDNA expressed specifically in omental adipose tissue, was a new adipocytokine identified by Schäffler et al. [37]. Omentin has been reported to enhance insulin-mediated glucose-uptake in adipocytes and to activate protein kinase Akt/PKB, which was also named insulin sensitizer [38, 39]. It is well known that immune vasculitis is the most characteristic pathologic change in KD patients, especially targeting coronary arteries among small and medium vessels. It has been conducted that the omentin-1 levels in synovial joints of patients with rheumatoid arthritis(RA) were lower than those of patients with osteoarthritis(OA) [38]. Patients with obesity showed decreased omentin-1 levels compared with thin patients and they suggested that omentin-1 levels may be predictive of the metabolic consequences or co-morbidities associated with obesity [39, 40]. In this study, we found that serum levels of omentin-1 as a kind of anti-inflammatory adipokine decreased in the acute stage of patients with KD, which could be explained that omentin-1 as a protective factor might play vital roles in anti-inflammation and inhibition the activation of endothelial cells at lower levels. What’s more interesting is that we found circulating omentin-1 levels are positively correlated with total cholesterol, which is a crucial biomarker of lipid metabolism. Lower omentin-1 may be beneficial to correct the disorders of lipid metabolism through inhibiting oxLDL-induced foam cell formation and protecting vascular endothelial cells from inflammatory lesions and alleviating vascular injuries of KD [41]. However, Antonella et al. [19] reported that serum omentin levels were higher in KD patients than healthy controls. This result is just inconsistent with our results. This difference may be explained through the following three possible reasons. Firstly, omentin has two different subtypes, which might play different roles in the development of KD. In this study, we focus on the correlation between omentin-1 and acute KD. But it is not clear Antonella and his or her colleague studied which subtype or total in their article. Secondly, we found that omentin-1 in the KD group was lower than febrile controls but not than healthy controls. However, Antonella et al. reported that serum omentin levels were significantly higher in KD patients versus healthy controls. Lastly, we have to take individual differences from different regions into account. In short, these controversial results need to be further confirmed by numerous studies.

Adiponectin is the most abundant adipokine secreted by adipocytes, which exhibits multiple physiological functions through combined to its receptors. In vitro and vivo experiments showed that adiponectin treatment attenuates lipopolysaccharide (LPS)-induced expression of TNF-a in cultured macrophages through inhibition of NF-κB signaling and overexpression of adiponectin alleviates progression of atherosclerotic lesions in apolipoprotein E knockout (KO) mice, with an accompanying decrease in TNF-a and SR-A expression [42, 43]. Here, this research indicates that adiponectin levels reduced significantly in the acute phase of KD, as with omentin-1 levels. The possible pathogenesis might be that adiponectin plays anti-inflammatory roles through blockade of NF-κB signaling and further inhibits the production of proinflammatory cytokines such as TNF-α and MMP-12 [44, 45]. It is worth noting that TNF-α is still overexpressed in the acute phase of KD might because that it has exceeded the inhibition properties of adiponectin though this still needs to be explored through in vitro and vivo experiments. In addition, we didn’t discover any pieces of evidence to support the correlation between adiponectin and coronary artery abnormalities of KD.

Recently, there were some case reports and a clinical trial reported that IL-1 receptor inhibitors (IL-1RA) improve the pathogenetic condition of KD with the restoration of dilation coronary artery [46–49]. We found that a low level of IL-1β in KD patients accompanied coronary artery lesions(CAL). This suggests that IL-1β is involved in the progress of coronary artery lesions, which implied that IL-1βbecome a new target in the treatment of KD or KD accompanied CAL in the future. This study provides evidence for this conclusion.

Ishwarlal et al. reported that lower levels of omentin-1 and higher levels of chemerin in nascent metabolic syndrome were risk factors for diabetes and cardiovascular disease [50]. In this study, disorders of lipid metabolism are present in patients with acute KD, which is consistent with the results reported previously [51]. We also found that serum levels of omentin-1 were positively correlated with both chemerin and total cholesterol, which suggests that chemerin and omentin-1 are directly or indirectly closely related to disorders of lipid metabolism in the acute phase of KD. Adipokines were involved in lipid metabolism in autocrine or paracrine manners and these disorders of lipids metabolism may be associated with inappropriate secretion of omentin-1 and chemerin which suggests that low levels of omentin-1 and chemerin may predict disorders of lipid metabolism, although this still needs to be supported by a larger data sample. Interestingly, it showed that hemoglobin in acute KD was higher than febrile control, which implied that hemoglobin was related to inflammation state in acute KD. Kim et al. [18]. described that resistin and serum IL-6 were significantly elevated and hemoglobin significantly lower in KD patients with coronary anomalies. Hemoglobin levels were negatively correlated with resistin levels in KD patients. However, it’s a pity that we didn’t find the correlation between hemoglobin and adipokines including chemerin, omentin-1, and adiponectin.

However, our study has several limitations. Firstly, we concentrated on the influences of each adipokine on KD respectively but cannot evaluate the interrelationship among different adipokines. Moreover, we only analyzed levels of adipokines including chemerin, omentin-1 and adiponectin in the acute stage without tracking the changing process of adipokines during the full development of KD and without further studying mechanism of action. Finally, our data are confined to a limited number of samples and concentricity of the region, thus further research with a larger sample and covering multi-centers will be more persuasive.

## Conclusions

Overall, we demonstrated that high chemerin, low omentin-1 and adiponectin levels are present in the acute stage of KD, suggesting that adipokines chemerin, omentin-1, and adiponectin are involved in acute KD. Furthermore, though the mechanism is unknown, chemerin might play pro-inflammatory, while omentin-1 and adiponectin may play an anti-inflammatory role in inflammation of acute KD. Although it didn’t show any relationship between there adipokines and coronary artery abnormalities of KD, we found that chemerin and omentin-1directly or indirectly related to lipid metabolism disorders. This provides new ideas for exploring the pathogenesis and treatment of refractory KD. It will be more interesting and meaningful to study how different isoforms of these adipokines work in inflammation and the relationship between hemoglobin and other adipokines in the acute phase of KD.

## Abbreviations

AOA: Aortic ring diameter
CAL: Coronary artery lesions
CRP: High sensitivity c-reactive protein
ESR: Erythrocyte sedimentation rate
HB: Hemoglobin
HDL: High-density lipoprotein
IVIG: Intravenous immunoglobulin
KD: Kawasaki disease
LCA: Left coronary artery
LDL: Low-density lipoprotein
NCAL: No coronary artery lesion group
PCT: Procalcitonin
PLT: Platelet
RCA: Right coronary artery
TC: Total cholesterol
TG: Triglyceride
